# A new bacteriophage infecting *Staphylococcus epidermidis* with potential for removing biofilms by combination with chimeric lysin CHAPSH3b and vancomycin

**DOI:** 10.1128/msphere.01014-24

**Published:** 2025-02-21

**Authors:** Ana Catarina Duarte, Lucía Fernández, Ana Rodríguez, Pilar García

**Affiliations:** 1 Instituto de Productos Lácteos de Asturias (IPLA-CSIC), Oviedo, Asturias, Spain; 2 DairySafe Group, Instituto de Investigación Sanitaria del Principado de Asturias (ISPA), Oviedo, Spain; University of Galway, Galway, Ireland

**Keywords:** *Staphylococcus epidermidis*, bacteriophage, biofilms, lytic protein, antibiotic, synergy

## Abstract

*Staphylococcus epidermidis* is the cause of serious skin and prosthetic joint infections despite being a common inhabitant of human body surfaces. However, both the rise in antibiotic resistance in this species and its ability to form biofilms are increasingly limiting the available therapeutic options against these illnesses. In this landscape, phage therapy stands out as an interesting alternative to antibiotics. In the present study, we report the isolation and characterization of a novel virulent phage infecting *S. epidermidis* (*Staphylococcus* phage IPLA-AICAT), which belongs to the *Herelleviridae* family. The estimated genome size of this virus is 139,941 bp, and sequence analysis demonstrated the absence of antibiotic resistance genes and virulence factors. This phage infects a high proportion (79%) of clinically relevant *S. epidermidis* strains and exhibits antibiofilm activity. Moreover, a combination of this phage with other antimicrobials, i.e., vancomycin and the lytic protein CHAPSH3b, further improved the reduction in surface-attached bacteria. Notably, the combination of *Staphylococcus* phage IPLA-AICAT (10^9^ PFU/mL) and CHAPSH3b (8 µM), originally designed to target *Staphylococcus aureus*, was able to reduce the number of viable cells by 3.06 log units in 5-h-old biofilms. In 24-h-old biofilms, the reduction was also significant after 6 h of treatment (2.06 log units) and 24 h of treatment (2.52 log units). These results confirm our previous data regarding the potential of phage-lysin mixtures against staphylococcal biofilms.

**IMPORTANCE:**

*Staphylococcus epidermidis* is one of the main causes to device-associated infections mostly due to its ability to form stable biofilms attached to human tissues. Besides the inherent antimicrobial tolerance of biofilms, this microorganism is also increasingly becoming resistant to standard-of-care antibiotics. To fight against this problem, phage therapy is a viable option to complement the available antibiotics in the treatment of recalcitrant infections. This work describes a new phage infecting *S. epidermidis* clinical strains that is a member of the *Herelleviridae* family and the combination with other antimicrobials to further improve the reduction of biofilms. Together with the significant progress achieved in the development of diagnostic tools, phages and their derived proteins will bring us much closer to a therapeutic landscape in which we are not so heavily reliant on antibiotics to combat bacterial pathogens.

## INTRODUCTION

The gradual increase in antibiotic resistance in bacteria is one of the biggest threats that we are facing today, affecting not only human health but also the global economy. Some experts estimate that the global economic burden of antimicrobial resistance could be in the trillions of dollars by 2050 (1). Increasingly, health care-associated infections represent one of the most common causes of morbidity and mortality worldwide, most frequently affecting immunocompromised or catheterized patients (2). Furthermore, since bacteria in these facilities are more commonly exposed to antibiotics, nosocomial strains tend to exhibit a higher prevalence of antibiotic resistance than those isolated in the community at large, making these infections more difficult to treat.

*Staphylococcus epidermidis* is one of the most abundant species in the human skin and mucosal microbiome. Indeed, colonization by this bacterium promotes skin barrier development, maintains homeostasis, actively coordinates the skin response to injury, and controls opportunistic pathogens through secretion of antimicrobial peptides (i.e., phenol soluble modulins) (3). Even though it may seem harmless for humans, this microorganism can behave as an opportunistic pathogen, mainly affecting hospitalized patients and premature infants (4). Worryingly, treatment of *S. epidermidis* infections is becoming challenging due to the increasing rates of antibiotic resistance in this species, even in strains isolated from healthy skin (5). Also, there is an increased prevalence of virulence genes in this pathogen, such as the extracellular cysteine protease EcpA, which has been related to skin diseases like severe atopic dermatitis (6). Apart from that, the biofilm-forming ability of this bacterium allows its colonization of medical devices and human organs (7). A biofilm is a surface-attached bacterial community embedded in an extracellular polymeric substance (EPS) secreted by the cells (8). This EPS is composed of polysaccharides, proteins, extracellular DNA (eDNA), and other biomolecules such as lipids (9). EPS promotes cell–cell cohesion (including interspecies recognition) to facilitate microbial aggregation and biofilm formation. Additionally, the physical and chemical properties of the biofilm matrix constituents, together with other properties of these complex communities, protect sessile cells from external challenges, such as antibiotics and host defense mechanisms. This makes the eradication of biofilms rather problematic, being the main reason of chronic recalcitrant infections. Thus, *S. epidermidis* is a leading cause of prosthetic valve endocarditis and implant-associated infections (10). This can be even worse when these biofilms disseminate into the cardiovascular system and cause potentially fatal bloodstream infections.

Over the last years, the interest in bacteriophages (phages) and their derived proteins as a potential alternative treatment for antibiotic-resistant bacterial infections has been on the rise. Phages are viruses that infect bacteria and have a narrow host range, being usually able to infect only one or a few species (11). Based on the successful results of clinical trials and case studies, more and more reports suggest the effectiveness of phage therapy, for example, against untreatable chronic infections (12) or periprosthetic joint infections (13). Moreover, bacteriophages have been tested as antibiofilm agents *in vitro* (14), both alone (individually or in phage cocktails) and in combination with other antimicrobials, such as antibiotics or antiseptics (15–17) and even with phage lytic enzymes (18).

To date, the number of identified phages that specifically infect *S. epidermidis* is still quite low (19–22). Among them, there are three siphoviruses (vB_SepiS-phiIPLA5, vB_SepiSphiIPLA6, and vB_SepiS-phiIPLA7) previously isolated and characterized in our laboratory (23). Genome sequencing of phages vB_SepiSphiIPLA5 and vB_SepiS-phiIPLA7 allowed the identification of genes encoding depolymerase activities against bacterial biofilms (24).

Additionally, we have identified and developed several phage lytic proteins with improved activity against staphylococci, including the chimeric protein CHAPSH3b (CHAP domain from the virion-associated protein HydH5 fused to the cell wall binding domain SH3b of lysostaphin) (25). CHAPSH3b not only demonstrated the ability to eliminate preformed structures but also inhibited biofilm development (26). Moreover, Duarte et al. reported the synergistic effects of combining CHAPSH3b with the virulent phage *Kayvirus* RODI in 24-h-old biofilms, resulting in a greater reduction in viable cell counts compared to the individual treatments (18). In this study, a new phage, named *Staphylococcus* phage IPLA-AICAT (AICAT), infecting *S. epidermidis* strains from clinical origin was isolated and characterized. Additionally, we tested the combination of this phage with other antimicrobials, the antibiotic vancomycin and the chimeric lytic protein CHAPSH3b, for biofilm removal.

## RESULTS

### Isolation and characterization of a new *S. epidermidis* phage

**Fig 1:**
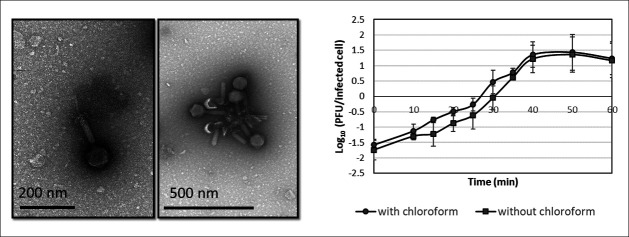
Transmission electron microphotographs and one-step growth curve of phage AICAT. Values correspond to the means ± standard deviations of four independent experiments represented in the number of PFUs per infected cell. Cells were chloroform treated (circles) or left untreated (squares).

Phage therapy is a promising strategy to fight against *S. epidermidis* infections resistant to current treatments; therefore, our aim was to isolate lytic phages and to investigate their future potential as therapeutics. For this purpose, 24 *S. epidermidis* strains from clinical origin were selected. Nine strains were isolated from bloodstream cultures, 10 were from breast milk, one was from a catheter, one was from a urine culture, two of them were with unknown origin, and one was a collection strain (from the blood of a patient with an intravascular catheter). Of note, all of the strains were selected considering their lysogenic pattern, using mitomycin C to check the presence/absence of prophages in their genome (27). After confirming the absence of prophages, eight strains were chosen arbitrarily and used to design four different mixtures, each of them composed by four strains, in order to maximize the chance of finding different phages. Enrichment cultures were performed using residual water, and after three enrichment steps, the supernatants were plaqued on all the strains showing the presence of lysis plaques with a different morphology. Depending on the transparency of the halo, the strains were considered more or less susceptible (Table 1). Taking into account the mixtures used for the enrichments (Table S1) and the susceptibility of the strains to the phages, strain SE11B was selected as a host strain for phage propagation. After three isolation rounds, which involved selecting and re-isolating specific lysis plaques each time to guarantee that there was only one phage in the suspension, we isolated a phage that was named *Staphylococcus* phage IPLA-AICAT (AICAT). The phage showed a wide host range, infecting 19 out of 24 *S*. *epidermidis* strains tested (79%) (Table 1). Only five strains (*S. epidermidis* SE1B, *S. epidermidis* SE8B, *S. epidermidis* SE16U, *S. epidermidis* 48, and *S. epidermidis* DG2ñ) were resistant, while the rest displayed different degrees of susceptibility. Six *S*. *aureus* strains were tested, and all of them were resistant to the phage. The morphology of the phage particles was observed by transmission electron microscopy (TEM). Virions had an icosahedral capsid, with a diameter of 73 ± 0.07 nm, and a long contractile tail, with a length of 97 ± 0.06 nm. The TEM images show that the phage has a hexagonal base plate with possible tail fibers. These characteristics indicate that phage AICAT is a myovirus belonging to the class *Caudoviricetes* (Fig. 1).

**TABLE 1:** Staphylococcal strains used in this work^*a*^

Strain	Origin	Reference	AICAT
*S. epidermidis* SE1B	Blood culture (San Agustín hospital)	This study	−
*S. epidermidis* SE2H	Blood culture (San Agustín hospital)	This study	+++
*S. epidermidis* SE3H	Blood culture (San Agustín hospital)	This study	++
*S. epidermidis* SE4B	Blood culture (San Agustín hospital)	This study	++
*S. epidermidis* SE3C	Catheter (San Agustín hospital)	This study	++
*S. epidermidis* SE6B	Blood culture (San Agustín hospital)	This study	+
*S. epidermidis* SE7B	Blood culture (San Agustín hospital)	This study	++
*S. epidermidis* SE8B	Blood culture (San Agustín hospital)	This study	−
*S. epidermidis* SE16U	Urine (San Agustín hospital)	This study	−
*S. epidermidis* SE10B	Blood culture (San Agustín hospital)	This study	+++
*S. epidermidis* SE11B	Blood culture (San Agustín hospital)	This study	+++
*S. epidermidis* 47	Unknown (San Agustín hospital)	This study	+++
*S. epidermidis* 48	Unknown (San Agustín hospital)	This study	−
*S. epidermidis* F12	Woman’s breast milk	(28)	+++
*S. epidermidis* CECT4183	Collection strain (blood of patient with intravascular catheter)	This study	+++
*S. epidermidis* ASLD1	Woman’s breast milk	(28)	++
*S. epidermidis* DG2ñ	Woman’s breast milk	(28)	−
*S. epidermidis* LO5081	Woman’s breast milk	(28)	+++
*S. epidermidis* LX5RB4	Woman’s breast milk	(28)	++
*S. epidermidis* Z2LDC14	Woman’s breast milk	(28)	+++
*S. epidermidis* YLIC13	Woman’s breast milk	(28)	+++
*S. epidermidis* DH3LIK	Woman’s breast milk	(28)	+++
*S. epidermidis* LO5RB1	Woman’s breast milk	(28)	++
*S. epidermidis* B	Woman’s breast milk	(28)	+++
*S. aureus* V329	Bovine subclinical mastitis	(29)	−
*S. aureus* BIM1	V329 derived mutant	(30)	−
*S. aureus* 15981	Clinical isolate	(31)	−
*S. aureus* Newman	Clinical isolate	(32)	−
*S. aureus* USA300 JE2	Clinical isolate	(32)	−
*S. aureus* IPLA16	Meat industry surface	(33)	−

^*a*^ Sensitivity of the strains to phage AICAT is indicated as follows: −, no inhibition halo (resistant); +, small halo (low susceptibility); ++, intermediate halo (medium susceptibility); +++, large halo (high susceptibility).

**Fig 2:**
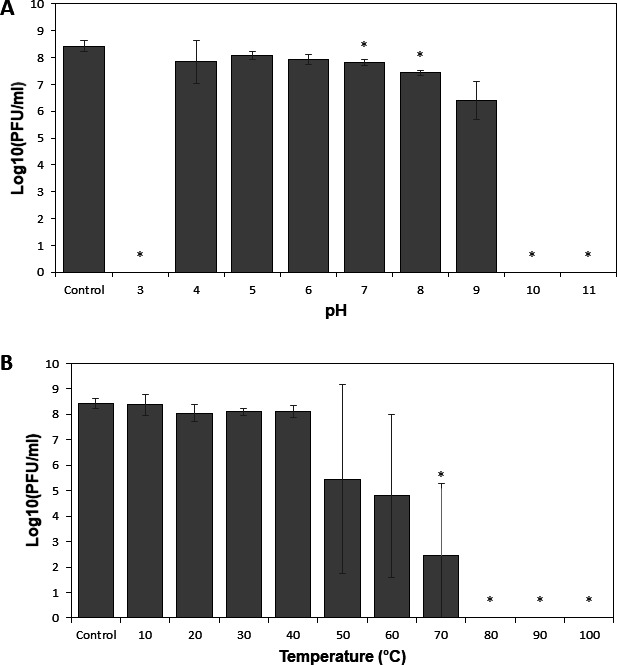
Stability of phage particles to environmental conditions, (**A**) pH and (**B**) temperature. Suspensions (10^8^ PFU/mL) of *Staphylococcus* phage AICAT were incubated for 30 min. Data correspond to the means ± standard deviations of three independent experiments and are represented in logarithmic scale in plaque-forming units per milliliter. Bars having an asterisk are significantly different (*P* < 0.05) from the control according to Student’s *t*-test.

The infection parameters of phage AICAT were determined by carrying out a one-step growth curve on strain *S. epidermidis* SE11B. As is observed in Fig. 1, the lytic cycle was around 40 min. Regarding the burst size, the estimated number of particles released per infected cell was 57. In terms of stability, this phage exhibited a high tolerance to pH, being stable in a range between pH 4 and pH 9. No viable infecting phages were recovered after incubation at pH 3 and pH 10 (Fig. 2A). Also, phage AICAT was very stable at temperatures below 50°C. Incubation at 70°C reduced the phage titer by 5.99 log units, whereas total inactivation was observed at 80°C (Fig. 2B).

### Determination of the lowest phage concentration that inhibits planktonic growth

**Fig 3:**
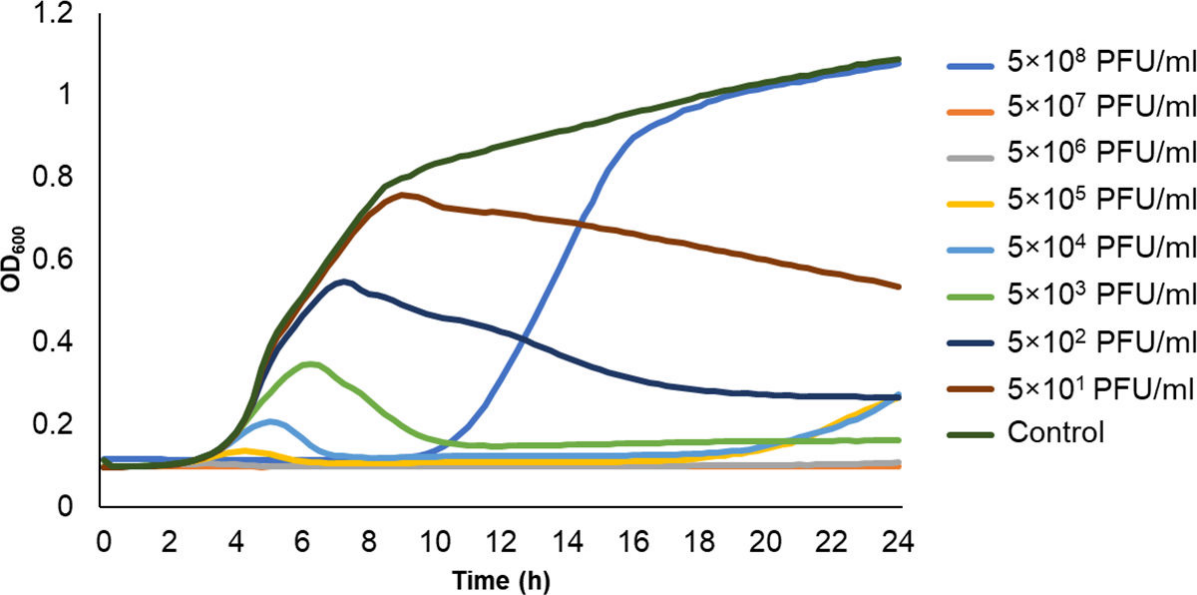
Growth curve of *S. epidermidis* SE11B at 37°C in the presence of increasing concentrations of phage AICAT ranging from 0 (control) to 5 × 10^8^ PFU/mL. OD_600_ was monitored for 24 hours. Data represent one representative experiment out of three independent replicates showing the same trend.

In order to evaluate susceptibility to phage AICAT, we performed a time-killing assay using the host strain SE11B. Phage was incubated at different concentrations with the bacteria during 24 h. The results show that the lowest starting phage concentration to inhibit bacterial growth was 5 × 10^3^ PFU/mL (Fig. 3). Lower starting concentrations did not sufficiently deplete the susceptible bacterial population, and regrowth of the bacterial population potentially due to the selection of bacteriophage-resistant mutants was observed at concentrations of 5 × 10^4^ PFU/mL, 5 × 10^5^ PFU/mL, and 5 × 10^8^ PFU/mL. No phage-resistant population was detected when using starting phage titers of 5 × 10^6^ PFU/mL and 5 × 10^7^ PFU/mL within the time frame of the experiment. Indeed, these concentrations seem to achieve total elimination of the bacterial population.

### Genome characterization of phage AICAT reveals it belongs to the genus *Sepunavirus*

The *Staphylococcus* phage IPLA-AICAT has a double-stranded DNA genome consisting of 139,941 bp carrying 206 putative open reading frames (ORFs) (accession number PQ589826). ORFs in the phage genome were annotated based on similarity to previously characterized *Staphylococcus* phage vB_SepM_BE04 (accession number MT596501). The morphogenesis module was split into two main regions in both genomes, which were separated by the replication/transcription module. Genes encoding the large and small terminase subunits, portal protein, prohead protease, major capsid, major tail sheath, tail fiber, tail baseplate, and tape measure protein were identified. A group I intron associated with a VRS endonuclease was detected in the middle of the terminase large subunit gene (*orf*65, *orf*66, *orf*67, and *orf*69). A lysis module, containing genes involved in bacterial lysis (holin and endolysin), was located upstream of the morphogenetic module. In addition, a second endolysin and holin genes were identified individually in the replication region. Putative lytic transglycosylase (*orf*97), amidase (*orf*98), and endolysin (*orf*96) genes were identified in the structural module, which may be involved in cell wall hydrolysis necessary for phage infection.

**Fig 4:**
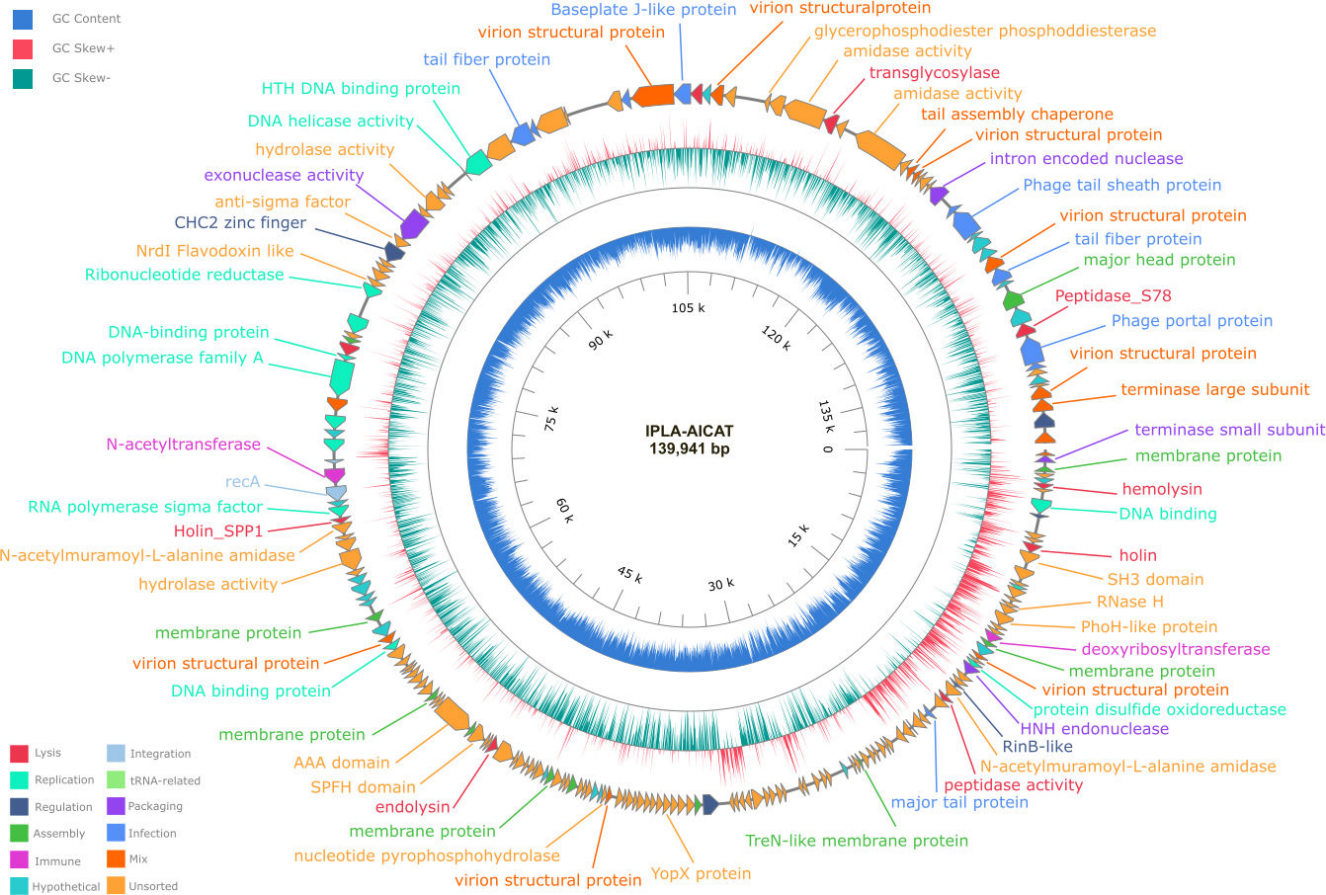
Genome organization of phage AICAT. The outer ring with arrows represents the ORFs of the circularized phage. The predicted genes functions are also indicated. The GC content is in the blue ring, the red ring is the GC skew +, and the green ring is the GC skew –.

**Fig 5:**
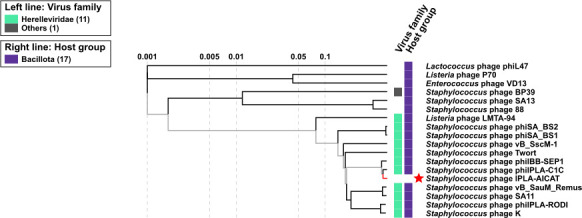
Viral proteomic tree. Phylogenetic tree created using VIPtree. The tree is drawn to scale, with branch lengths measured represented in logarithmic scale. Phage AICAT, highlighted with a red star, was compared with other phages infecting members of to the phylum Bacillota.

At the nucleotide level, phage AICAT shares a high degree of similarity with *Staphylococcus* virus BESEP4 (93.88%) and 92.50% with the *Staphylococcus* phage 80A, being a new species belonging to genus *Sepunavirus*. Additionally, it was closely related to phages 110 (91.44%), phiIPLAC1C (90.43%), twinlingate (88.87%), and Quidividi (86.37%), according to VIRIDIC. The online database PhageScope was used to perform the comprehensive annotation of phage AICAT including the morphogenesis, replication/transcription, long terminal repeat, and lysis modules (Fig. 4), and VIPtree software was used for the generation of the proteomic tree to reveal the similarity relationship with other virus (Fig. 5).

### Phage susceptibility of biofilms might be related to matrix composition

**Fig 6:**
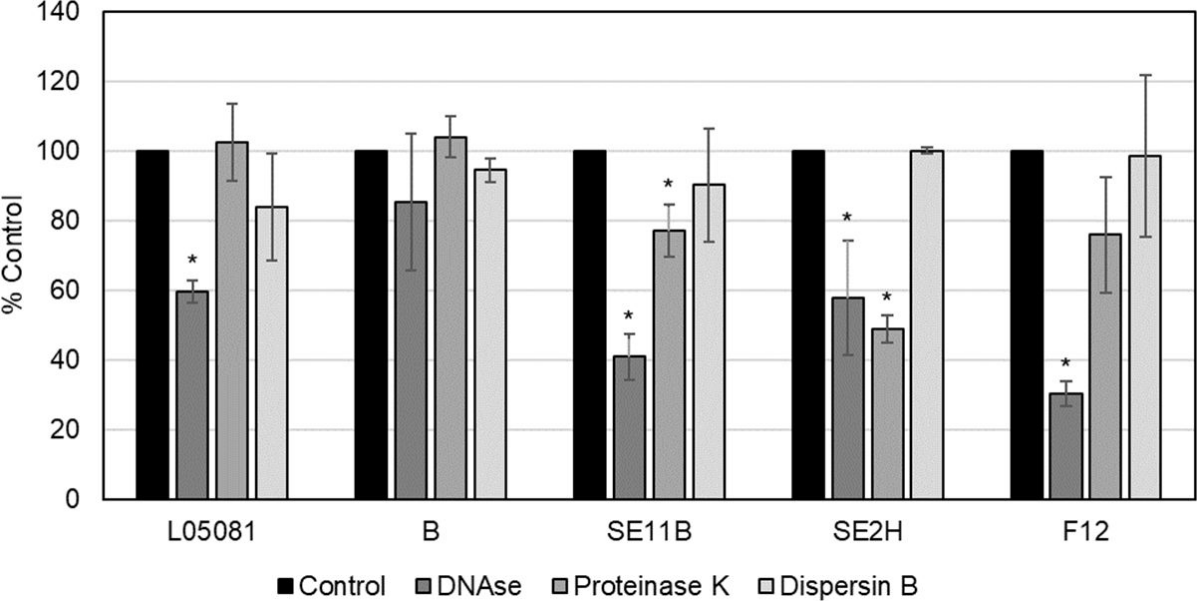
Chemical composition of the extracellular matrix of *S. epidermidis* biofilms. The 24-h-old biofilms were treated with DNase, proteinase K, and dispersin B, and the attached biomass was measured and compared to control samples.

The design of a phage-based antibiofilm strategy against *S. epidermidis* would benefit from knowing the composition and structure of the target biofilms. With this in mind, we selected five *S*. *epidermidis* strains to determine the composition of their biofilm matrix: *S. epidermidis* SE11B and *S. epidermidis* SE2H because of their high sensitivity to the new phage, as well as *S. epidermidis* F12, *S. epidermidis* L05081, and *S. epidermidis* B due to our previous knowledge about their phage sensitivity to other phages (34). Treatment of 24-h-old biofilm samples with degrading enzymes (DNase, proteinase K, and dispersin B) indicated that the biofilm matrix of strains *S. epidermidis* L05081, SE11B, SE2H, and F12 was mostly composed of eDNA. Interestingly, *S. epidermidis* strains SE11B and SE2H also contained a significant amount of proteins (Fig. 6).

**Fig 7:**
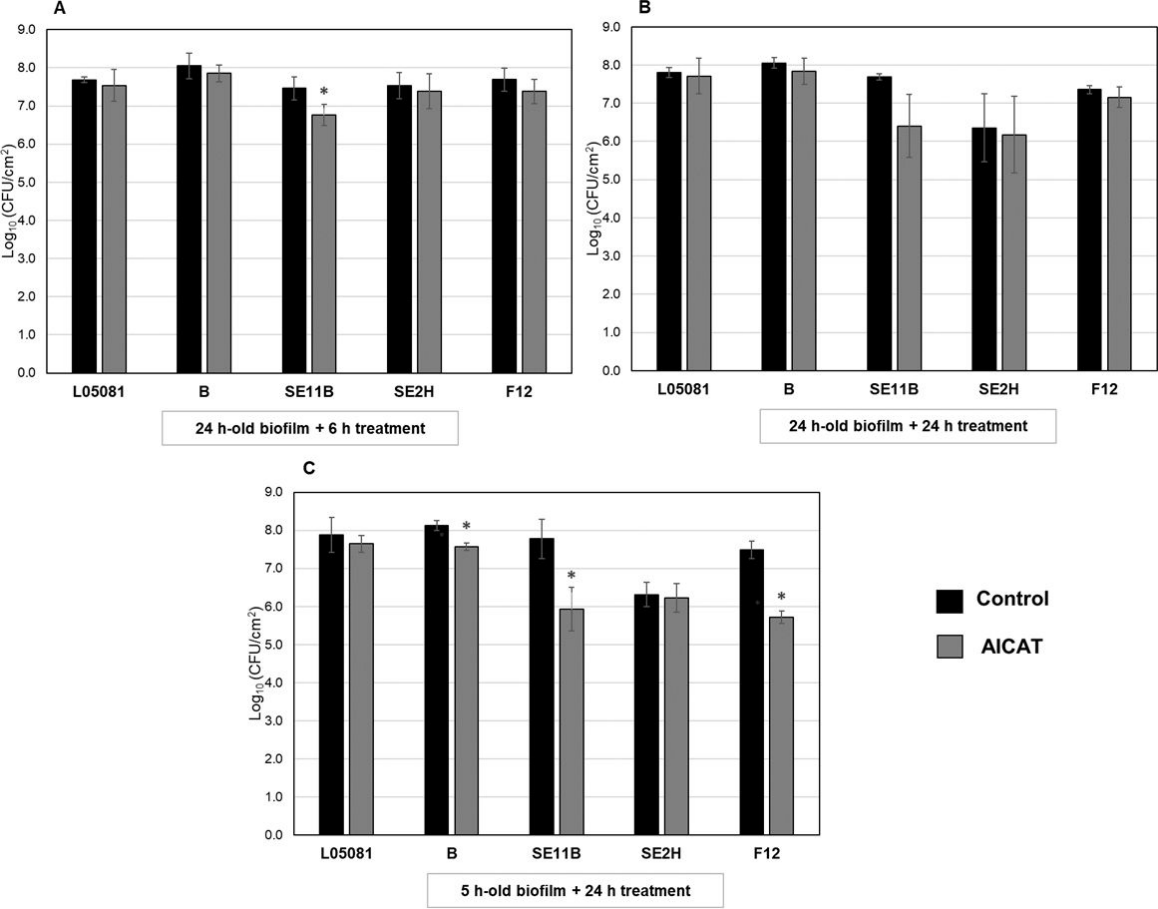
Treatment of biofilms formed by different *S. epidermidis* strains. Biofilms were allowed to develop during 24 h at 37°C and treated during 6 h (**A**) and 24 h (**B**) and 5 h treated 24 h (**C**) with phage AICAT (dark gray bars). The tryptic soy broth (TSB) medium alone was added to the control wells (black bars). After incubation, the viable cell counts of the five strains were determined. Data correspond to the means ± standard deviations of three independent experiments and represented in the logarithmic scale in colony-forming units per unit area of attachment surface. Bars with an asterisk are statistically different (*P* < 0.05) from the untreated control according to Student’s *t*-test.

The use of strains with different biofilm matrix composition provides us with a variety of targets to test antibiofilm efficacy. The 24-h-old biofilms formed by these strains were treated individually with the phage (10^9^ PFU/mL) for 6 h or 24 h (Fig. 7A and B). When 24-h-old biofilms were infected with AICAT for 6 h, significant reductions in CFUs were only observed for *S. epidermidis* strain SE11B (0.69 log unit reduction) (Fig. 7A). Apart from that, phage AICAT was not able to reduce viable cell counts in any other strain. The low sensitivity of biofilms to this phage led us to increase the infection time to 24 h. Nonetheless, only strain *S. epidermidis* SE11B was sensitive, with an average reduction of 1.28 log units, although this difference was not statistically significant (Fig. 7B).

Early biofilms were also treated with phage AICAT. Interestingly, in 5-h-old biofilms (Fig. 7C), there was a significant reduction in viable cells for strains *S. epidermidis* B, *S. epidermidis* SE11B, and *S. epidermidis* F12 (0.56, 1.84, and 1.77 log units, respectively) after 24 h of treatment. Overall, *Staphylococcus* phage AICAT seems to be effective, especially against *S. epidermidis* SE11B and *S. epidermidis* F12, which happen to possess a matrix with a high eDNA content. Nonetheless, it is difficult to establish a correlation between matrix composition and phage susceptibility based on such a small number of strains.

### Combined treatment improves biofilm removal

**Fig 8:**
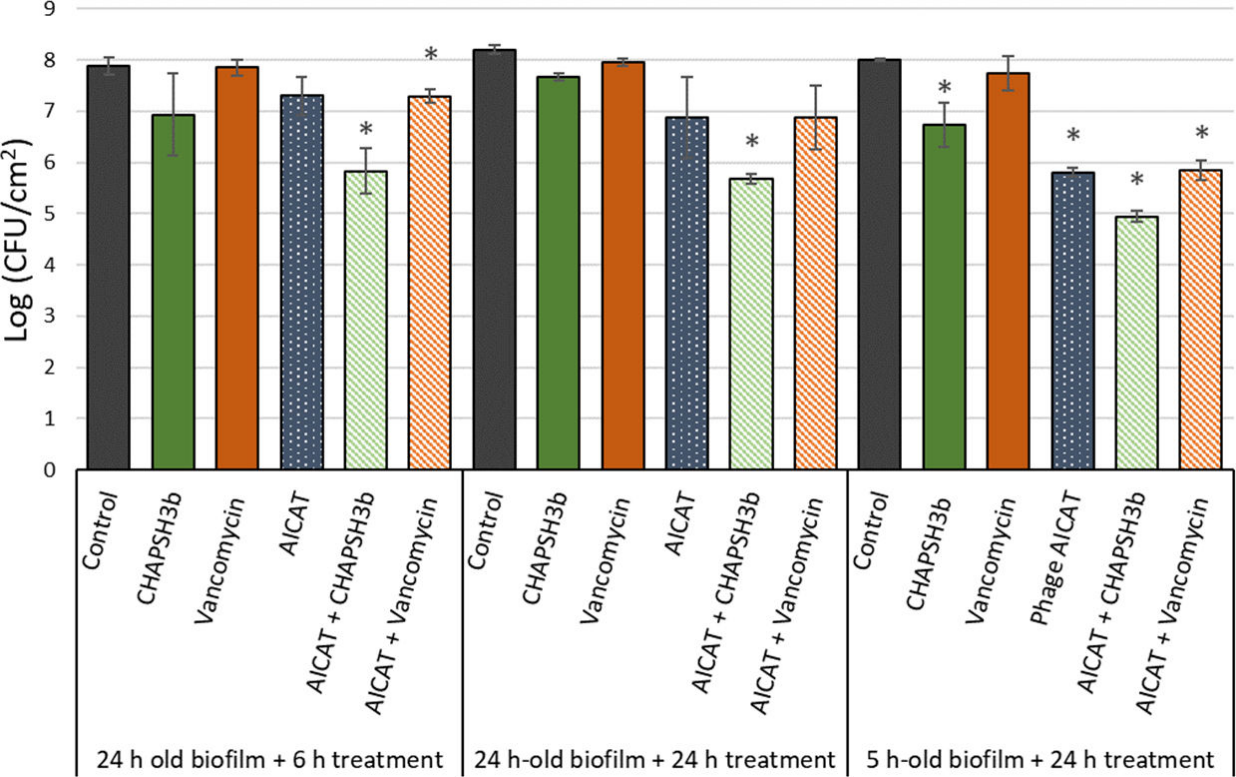
Combined treatment of phage AICAT with different antimicrobials against *S. epidermidis* SE11B biofilms. Biofilms (5 h and 24 h old) were treated (6 h and 24 h) with phage AICAT (10^9^ PFU/mL), chimeric protein CHAPSH3b (8 µM), vancomycin (4 µg/mL), or combinations of the phage with the antimicrobials. Fresh TSB medium was added to the control wells. After incubation, the viable cell counts were determined. Data correspond to the means ± standard deviations of three independent experiments and are represented in the logarithmic scale in colony-forming units per square centimeter of attachment surface. Bars with an asterisk are statistically different (*P* < 0.05) from the untreated control according to Student’s *t*-test.

Given the poor results obtained with the phage alone, we combined phage AICAT (10^9^ PFU/mL) with other antimicrobials, namely, the lytic protein CHAPSH3b (8 µM) and vancomycin (4 µg/mL). The results demonstrated that the combination of the phage with CHAPSH3b works significantly better (decrease of 2.52 log units) than the phage alone after 24 h of treatment in 24 h biofilms (Fig. 8). These results indicated a synergistic effect between phage AICAT and the lytic protein with an interaction index of 0.65. In contrast, there was an additive effect in the combination of the phage with vancomycin, with an interaction index of −0.25, even though the reduction in viable cells using the phage-antibiotic combination was similar to that obtained for the phage alone (1.33 log units), and there was no statistically significant reduction in the biofilms compared with the untreated control. No significant reduction in viable cells was observed when using the lytic protein or the antibiotic alone (Fig. 8).

When treatment was implemented only for 6 h in 24 h biofilms, the best combination was the phage with the protein CHAPSH3b with a reduction of 2.06 log units, acting synergistically with an interaction index of 0.50. The combination of the phage with the antibiotic resulted in an additive effect, with an interaction index of −0.03.

This assay was also performed with 5-h-old biofilms (Fig. 8). Again, the combination of phage and CHAPSH3b gave the best results in terms of biofilm reduction (decrease of 3.06 units log) followed by combination of phage with vancomycin (2.16 log units). However, these results reflect an additive effect and not synergy, with an interaction index of −0.42 for CHAPSH3b and of −0.32 for the antibiotic.

## DISCUSSION

The ability of *S. epidermidis* to cause device-associated infections is mainly due to its ability to form stable biofilms attached to human tissues. Besides the inherent antimicrobial tolerance of biofilms, this microorganism is also becoming increasingly resistant to standard-of-care antibiotics. In this scenario, phage therapy is a viable option to complement the available antibiotics in the treatment of recalcitrant infections. Indeed, it is experiencing significant momentum as demonstrated by the fact that several clinical trials are currently under way. Some examples include safety studies of bacteriophages (ClinicalTrials.gov ID NCT04650607) and therapy in patients with diabetic foot osteomyelitis (ClinicalTrials.gov ID NCT05177107). Phages intended for the treatment of patients must comply with several requirements, such as being strictly virulent, lacking virulence factors and antibiotic resistance genes, and should not be prone to generalized transduction (35). Even though there are several ongoing studies, the number of identified virulent phages infecting this species is relatively small compared to those against *S. aureus*, and data regarding their antibiofilm efficacy remain scarce (36, 37). In this context, we have isolated a new phage infecting a number of *S. epidermidis* strains isolated from clinical samples, including some methicillin-resistant isolates. The characterization of phage AICAT revealed that it belongs to the *Herelleviridae* family, whose members are obligatorily lytic (https://ictv.global/report/chapter/herelleviridae/herelleviridae). Also, based on its similarity to other phages in the databases, phage AICAT is a new species belonging to the genus *Sepunavirus*. The phage genome confirmed that it does not carry any genes related to virulence, antibiotic resistance, or lysogeny, making this phage suitable for therapeutic purposes (35). Additionally, microscopy and bioinformatic analysis revealed characteristics consistent with other members of the *Herelleviridae* family, such as virions with myovirus morphology, double-stranded DNA (dsDNA) genomes of 125–170 kbp, and virulent (https://ictv.global/report/chapter/herelleviridae/herelleviridae). Moreover, other characteristics found in staphylococcal phages belonging to this family include the presence of homing endonucleases within group I introns, in accordance with previous results reported for other myophages, such as T4, where 11% of the ORFs correspond to homing endonucleases (38).

Besides host range analysis, we evaluated the antibacterial activity, stability, and infection parameters of this phage. Our results showed a high production of phage particles for phage AICAT (57 per infected cell) but a low stability at high pH. Taking into account that the pH in healthy skin is between 4.7 and 5.75, and between 7.15–8.9 in chronic wounds, this phage will be stable enough for therapeutic applications in skin-related infections. In any case, the issue of improving phage stability for their application in the treatment of wound infections has already been addressed with different solutions that include encapsulation or the design of materials that allow controlled release and protection of viral particles (39, 40).

Biofilm formation is critical for the virulence of *S. epidermidis* and the principal microbial property leading to the observed outcomes in periprosthetic joint infection management and device-related infections. In our work, biofilms formed by five different *S. epidermidis* strains were resistant to phage attack regardless of the strain origin. Similar results were observed by Melo et al., who concluded that the extracellular matrix is a barrier that hampers infection of bacteria rather than inactivate the phages or prevent phage adsorption (36). Despite the potential advantages of phage therapy, the complete eradication of biofilms by phages has not been demonstrated in literature to date; however, prevention and control of biofilm formation has been achieved for *S. aureus* biofilms, using phage K and modified derivatives (41) and *S. epidermidis* biofilms on catheter surfaces by using a phage pretreatment step. In this case, phage 456 could reduce growth of biofilms on hydrogel-coated and serum/hydrogel-coated silicone catheters (42). Overall, even though the use of our phage did not lead to a higher reduction in the biofilm, we did observe that younger biofilms are more sensitive to the phages individually and in combination with the antimicrobials. Moreover, *S. epidermidis* SE11B and *S. epidermidis* F12 were the most susceptible strains to phage attack. Interestingly, these strains have a high content of eDNA in the extracellular matrix, which is an important component during biofilm maturation. Also, DNA release from cells is critical for biofilm attachment during the initial stages of development in *S. aureus* (43) and *S. epidermidis* (44)*,* being mediated by the autolysins AtlA and AtlE, respectively. Regarding biofilm treatment with phage, it is not clear how eDNA interacts with the virions and facilitates infection by *Staphylococcus* phage AICAT. Perhaps, the anionic charge of eDNA may repel phage particles favoring diffusion. By contrast, the polysaccharide intercellular adhesin PIA, which has a net positive charge, may promote interactions between phage particles with components of the biofilm extracellular matrix or the staphylococcal cell wall, thereby hindering infection (45). A previous study described that biofilm matrix composition affects the sensitivity of staphylococci to disinfectants by reducing the diffusion and/or neutralizing the compounds (46). The efficacy of phages as clinical therapeutics depends on several factors, including the development of phage-resistant bacteria, pharmacokinetic complexity, and any potential human immune response. To date, there are no studies reporting total biofilm eradication using only phages; in fact most times, the treatment of patients with phages requires the combination of phage therapy and standard-of-care antibiotic treatment as an antibiofilm strategy. This combination has several advantages such as avoiding the development of phage resistance (47). Indeed, the treatment of *S. aureus* strains with phage SA11 and subinhibitory concentrations of antibiotics was found to be synergistic in inhibiting bacterial growth (15). The use of phages and vancomycin to remove biofilms has also been tested in *S. aureus*. The 48-h-old biofilms were treated for 72 h with phage K (10^9^ PFU), vancomycin (42 µg/mL), or a combination of both showing a synergistic activity (17), and Taha et al. demonstrated that combining phage Remus and vancomycin led to synergistic interaction against methicillin-resistant *S. aureus* biofilm-like aggregates *in vitro* and *in vivo* (48). To the best of our knowledge, there is only one study combining phages with vancomycin against S. *epidermidis* biofilms; however, they could not detect any synergistic effect between antibiotic and phage due to the high variability of the experimental technique (49). In our study, we also combined phage AICAT with vancomycin, an antibiotic commonly used to treat infections by methicillin-resistant staphylococcal strains. Even though we did not observe a synergistic interaction between the phage and the antibiotic under any of the conditions tested, there were no antagonistic interactions either. In contrast, Dickey et al. showed that vancomycin exerted an antagonistic effect when used in combination with some *S. aureus* phages (50). Another study by Tkhilaishvili et al. showed no synergistic effect with vancomycin when administrated simultaneously (51). Despite the lack of synergy observed in the combination of phage with vancomycin, the presence of the two antimicrobials will limit the development of resistance to the individual compounds, constituting an additional advantage. Furthermore, the fact that young biofilms are more sensitive to the action of the phage but not to the antibiotic can be exploited when designing a sequential treatment of phages and vancomycin. Interestingly, we did observe synergy when using a combination of AICAT with a phage-derived lytic protein. This is in good agreement with our previous results using phage *Kayvirus rodi* and the chimeric protein CHAPSH3b for *S. aureus* biofilm elimination (18). The potential of endolysins as adjuvants against biofilms is still poorly explored, although recent studies showed that the application of endolysins and phage exopolysaccharide depolymerase could increase antibiotic susceptibility and decrease cross-resistance to antibiotics (52). Also, the combination of endolysins and antimicrobial peptides may be a potential antimicrobial strategy for combating *Enterococcus faecalis* biofilms (53).

It must also be noted that the lesser impact of phage AICAT on biofilms compared to planktonic cells reflects not only the greater resilience of these structures to antimicrobials but also the challenges associated with biofilm-handling techniques (54). The complexity of these microbial communities together with limitations in their *in vitro* manipulation, such as high variation between replicates, often mask the potential efficacy of antibiofilm treatments.

The data obtained in this study support the potential of the virulent phage AICAT in the context of phage therapy, having a wide host range against *S. epidermidis* strains and demonstrating biofilm removal properties. However, its efficacy is still limited, even when combined with the antibiotic vancomycin, making it necessary to explore additional combinations with other phages or antimicrobials. The results obtained by treating biofilms with AICAT and the lytic protein CHAPSH3b were more promising, perhaps outlining the direction to be followed in future research. Moreover, the present results also highlight that more studies are still needed to fully understand the infection dynamics of phage AICAT in biofilms in order to maximize its potential.

## MATERIALS AND METHODS

### Bacterial strains, growth conditions, and protein purification

For this study, 24 *S*. *epidermidis* and six *S*. *aureus* strains were used (Table 1). Bacteria were grown at 37°C in tryptic soy broth (TSB; Scharlau, Barcelona, Spain) or in TSB plates with 1.5% (wt/vol) agar (Roko, S.A., Llanera, Spain) (TSA). TSB top agar composed of TSB supplemented with 0.7% (wt/vol) agar was used for phage propagation and titration. For biofilm formation assays, TSB supplemented with 0.25% (vol/vol) glucose (Merck, Darmstadt, Germany) (TSBg) was used.

The phage-derived protein CHAPSH3b and dispersin B were purified as described previously (55). The proteins were then checked by SDS-PAGE analysis and quantified by using the Quick Start Bradford Protein Assay kit (Bio-Rad).

### Phage isolation, purification, and titration

Bacteriophage was isolated from samples coming from a sewage treatment plant in Oviedo (Asturias, Spain). Two liters of sewage was centrifuged twice during 30 min at 1,000 rpm and 4°C. The supernatant was filtered by sequentially using cellulose acetate filters with 0.45 µm and 0.22 µm pore size membrane (VWR, Spain). The enrichment protocol for phage isolation was carried out using four mixtures composed each by four different *S. epidermidis* strains (Table S1). A total of 100 µL of each strain, previously grown to an OD_600_ ~0.1, was added to 80 mL of filtered sewage and 20 mL of 5 × TSB medium and grown overnight at 37°C with shaking. On the next day, these cultures were centrifuged and filtered. Three sequential enrichments were carried out to obtain a higher phage titer. To detect the presence of phages in the enrichment cultures, we dropped 5 and 10 µL of the supernatant from each mixture onto TSA plates containing each of the 24 *S*. *epidermidis* strains using the double layer technique (23). The presence of phages infecting these strains was determined by visualization of an inhibition halo after overnight incubation of these plates at 37°C. *S. epidermidis* SE11B was selected as host strain for plaquing culture supernatants. One isolated lysis plaque was taken, resuspended individually in SM buffer and plated. From this plate, a new isolated lysis plaque was taken again, and the process was repeated two more times. From the last round, several individual lysis plaques were selected for propagation as described previously (56). Phage enumeration was performed by the double layer technique (23).

### One-step growth curve

One-step growth curves were performed to determine the phage growth parameters using the sensitive strain *S. epidermidis* SE11B. First, 10 mL of a mid-exponential phase culture with an OD_600_ of 0.1 was harvested by centrifugation (4,000 rpm, 4°C, 10 min) and resuspended in 1 mL of fresh TSB medium. Phage was added to this suspension at an MOI (multiplicity of infection) of 1 and allowed to adsorb for 10 min at 37°C. Next, the mixtures were centrifuged again (4,000 rpm, 4°C, 10 min), and the pellet was resuspended in 10 mL of fresh TSB medium. A sample was taken immediately, and the suspension was incubated at 37°C. Further samples were taken every 10 min over a period of 60 min. Samples were centrifuged (13,000 rpm, 1 min), and the supernatant was immediately serially diluted in SM buffer (20 mM Tris-HCl, 10 mM MgSO_4_, 10 mM CaCl_2_, 100 mM NaCl , pH 7.5). The pellet was resuspended again and treated with 1% (vol/vol) chloroform for 1 min (vortex), in order to release the phages inside the cells, and centrifuged again (13,000 rpm, 1 min). The supernatant was serial diluted in SM buffer for PFU determination as described above. From the one-step curves, the infection parameters of the phage (latent period and burst size) were determined. The burst size was calculated by dividing the total number of phage progeny produced during a single round of infection by the total number of infected cells.

### Time-killing assay

The time-killing assay was performed to find out the susceptibility of planktonic bacterial cells to phage infection using the microdilution assay. In summary, strain SE11B was grown until OD_600_ ~0.1 and diluted 1:10 (vol/vol). Phage AICAT was added at different MOIs into a 96-well polystyrene microtiter plate and mixed with the bacteria. In order to monitor the evolution of the bacterial population in the presence of increasing phage concentrations, we monitored growth for 24 h at 37°C by measuring the OD_600_ every 15 minutes using a multiwell plate reader Tecan Infinite M Nano (Tecan Trading AG, USA).

### pH and temperature stability assays

The pH stability of the phage particles was tested by incubation in Britton-Robinson pH universal buffer (150 mM KCl, 10 mM KH_2_PO_4_, 10 mM sodium citrate, and 10 mM H_3_BO_3_), with adjustment of the pH in the range from 3 to 11. A phage suspension (10^9^ PFU/mL) was diluted 1:10 in universal buffer and incubated for 3 h at room temperature. Similarly, the temperature stability of the phage particles was examined by incubation of the phage (10^9^ PFU/mL) in SM buffer at different temperatures ranging from 10°C to 100°C for 30 min. Afterwards, serial dilutions were performed for PFU determination. Phage stored at 4°C was used as a control.

### Transmission electron microscopy

Electron microscopic analysis was performed after negative staining of the phage particles using 2% uranyl acetate. Visualization was carried out using a JEOL 12.000 EXII transmission electron microscope (JEOL USA Inc., Peabody, MA).

### Phage DNA extraction, genome sequencing, and bioinformatics analysis

Phage DNA extraction started with the elimination of the extracellular bacterial DNA from the phage suspension (10^9^ PFU/mL). Briefly, 250 µL of phage suspension was incubated with benzonase (125 U) (Sigma), RNase cocktail (5 U) (ThermoFisher Scientific), DNase I (10 U) (Fermentas), and TurboDNAse (5 U) (Invitrogen) during 2 h at 37°C. After digestion, 1 vol of phenol:chloroform (3:1) was added to the phage sample and mixed for 1 min. Then, the samples were centrifuged for 2 min (10,000 rpm), the upper phase was collected and transferred into a new tube, and the same process was repeated. Next, 1 vol of chloroform was added to the sample, mixed for 1 min, and centrifuged (10,000 rpm, 5 min). The upper phase was recovered and, after adding 25 µL of 3 M sodium acetate and 625 µL of 100% ethanol, incubated at −80°C for 30 min. After that, the samples were centrifuged for 15 min at 4°C, and the pellet was washed with ethanol 70% and then washed again with ethanol 100%. The sample was dried, resuspended in milliQ water, and stored at −20°C. Genome sequencing of phage AICAT was carried out at FISABIO Sequencing and Bioinformatics Service (Valencia, Spain) using Illumina technology. The phage genome was annotated using tools from the Bacterial and Viral Bioinformatics Resource Center (BV-BRC) (57), Open Reading Frame Finder (https://www.ncbi.nlm.nih.gov/orffinder/), and PhageScope online software (58). VIPtree software was used for the generation of a proteomic tree (59). BLASTP was used to search for similar proteins. Putative tRNAs, antibiotic resistance genes, and virulence genes were predicted using tRNAscan-SE, ResFinder4.1, and VFDB (VFDB: Virulence Factor Database), respectively. Genomic comparison at the nucleotide level was made with BLASTN using the genome sequences available in public databases (NCBI) and VIRIDIC (60).

### Biofilm formation and anti-biofilm efficacy of phages

Overnight cultures of five *S*. *epidermidis* strains (L05081, B, SE11B, SE2H, and F12) were diluted 1:100 (vol/vol) in fresh TSBg medium. Then, 1 mL from each bacterial suspension was poured into each well of a 24-well polystyrene microtiter plate (Thermo Scientific, NunclonTM Delta Surface) and incubated for 5 h or 24 h at 37°C, under static conditions. Following incubation, the planktonic phase was removed and the biofilms was washed once with phosphate-buffered saline (PBS; 137 mM NaCl, 2.7 mM KCl, 10 mM Na_2_HPO_4_, and 2 mM KH_2_PO_4_ [pH 7.4]). The remaining adhered cells were treated by addition of 0.5 mL of TSB alone as a control or containing a suspension of phage (10^9^ PFU/mL), vancomycin (4 µg/mL) or protein CHAPSH3b (8 µM) individually, phage (10^9^ PFU/mL) plus vancomycin (4 µg/mL), or phage plus protein CHAPSH3b (8 µM). All samples were incubated during 24 h at 37°C. Subsequently, the planktonic phase was removed, and the biofilms were washed with PBS. The number of viable cells was determined using the spot test. Briefly, the biofilms were scraped and resuspended in 1 mL of PBS. Then, 10-fold serial dilutions were performed, and 10 µL from each suspension was placed onto TSA plates and allowed to dry. The results are presented in number of colony-forming units per unit area (CFU/cm^2^). The potential interaction between the antimicrobials (phage and lysin or phage and antibiotic) was calculated (61), and the values obtained were named interaction indices. The interaction was considered additive when this index was between −0.5 and 0.5, antagonistic when the value was less than −0.5, and synergistic when the value was >0.5.

To determine the composition of the biofilm matrix, we washed once the adhered cells from 24-h-old biofilms with PBS, and then treated them with 0.5 mL of DNase (100 µg/mL), proteinase K (100 µg/mL), or dispersin B (6 µM) solutions during 1 h at 37°C. Then, total biomass was quantified by performing the crystal violet staining assay (62). Briefly, 1 mL of 0.1% (wt/vol) crystal violet was added and incubated for 15 min and next washed once with water to remove the excess of crystal violet and solubilized by adding 33% (vol/vol) of acetic acid. The amount of dye was quantified by measuring absorbance at 595 nm (*A*_595_) using a Benchmark Plus Microplate Spectrophotometer (Bio-Rad Laboratories, Hercules, CA, USA).

### Statistical analysis

Statistical analysis of the results was carried out by performing Student’s *t*-test, and *P* values lower than 0.05 were considered significant.

### Biosafety statement framework

This study was conducted in accordance with all applicable biosafety regulations and guidelines. All experiments involving pathogenic microorganisms were performed under Biosafety Level 2 conditions in a facility certified by Ministry of Agriculture, Food and Environment (A/ES/12/I-19). Approval for the study was obtained from the CSIC Institutional Biosafety Committee. Risk mitigation measures, including the use of personal protective equipment and validated decontamination procedures, were implemented to ensure the safety of personnel and the environment.

## SUPPLEMENTAL MATERIAL

Table S1 msphere.01014-24-s0001.docx Mixtures of strains used for phage enrichments.

ASM does not own the copyrights to Supplemental Material that may be linked to, or accessed through, an article. The authors have granted ASM a non-exclusive, world-wide license to publish the Supplemental Material files. Please contact the corresponding author directly for reuse.
