# Supplementary material for: A new bacteriophage infecting Staphylococcus epidermidis with potential for removing biofilms by combination with chimeric lysin CHAPSH3b and vancomycin
Source: mSphere. 2025 Feb 21;10(3):e01014-24. doi: 10.1128/msphere.01014-24 (PMC11934314; doi:10.1128/msphere.01014-24)
Supplement: Table S1 — Mixtures of strains used for phage enrichments. [file msphere.01014-24-s0001.docx]

| **Mixture** | **Strains** |
| --- | --- |
| Mix 1 | *S. epidermidis* SE2H  *S. epidermidis* SE3H  *S. epidermidis* SE4B  *S. epidermidis* SE3C |
| Mix 2 | *S. epidermidis* SE6B  *S. epidermidis* SE16U  *S. epidermidis* SE11B  *S. epidermidis* 48 |
| Mix 3 | *S. epidermidis* SE2H  *S. epidermidis* SE3H  *S. epidermidis* SE11B  *S. epidermidis* 48 |
| Mix 4 | *S. epidermidis* SE4B  *S. epidermidis* SE3C  *S. epidermidis* SE6B  *S. epidermidis* SE16U |

**Supplementary table 1 - Mixtures of strains used for phage enrichments.**
